# Rescaled Local Interaction Simulation Approach for Shear Wave Propagation Modelling in Magnetic Resonance Elastography

**DOI:** 10.1155/2016/9343017

**Published:** 2016-01-13

**Authors:** Z. Hashemiyan, P. Packo, W. J. Staszewski, T. Uhl

**Affiliations:** Department of Robotics and Mechatronics, AGH University of Science and Technology, Al. Mickiewicza 30, 30-059 Krakow, Poland

## Abstract

Properties of soft biological tissues are increasingly used in medical diagnosis to detect various abnormalities, for example, in liver fibrosis or breast tumors. It is well known that mechanical stiffness of human organs can be obtained from organ responses to shear stress waves through Magnetic Resonance Elastography. The Local Interaction Simulation Approach is proposed for effective modelling of shear wave propagation in soft tissues. The results are validated using experimental data from Magnetic Resonance Elastography. These results show the potential of the method for shear wave propagation modelling in soft tissues. The major advantage of the proposed approach is a significant reduction of computational effort.

## 1. Introduction

Mechanical properties of tissues are one of the most significant indicators used for detection of various abnormalities in medical diagnosis. Tumors and other pathologies often exhibit values of elastic moduli that are significantly different from healthy tissues. It is well known that none of the classical medical approaches, such as Computed Tomography (CT), Magnetic Resonance Imaging (MRI), and Ultrasonography (US), are able to detect mechanical properties of tissues that are diagnosed by palpation [1, 2]. Elastography is used extensively in diagnostic applications (e.g., liver fibrosis or breast tumors detection [3–9]) due to flexibility and noninvasiveness. Since abnormal tissues are often stiffer than the normal ones, medical diagnosis can be achieved. Although the method was developed in the late 1980s [10–12] the major breakthrough came in the mid 1990s when a dynamic approach to elastography was proposed [13]. A Motion-Encoding Gradient (MEG) was introduced to a conventional MRI system leading to Magnetic Resonance Elastography (MRE) [13–17].

Modelling in elastography relies on direct and inverse problems. The former relates to measurements of tissue responses to applied stresses. The latter is related to estimation of unknown mechanical properties from measured mechanical responses. Both problems are formulated using physical laws, which provide equations that relate biomechanical properties, such as shear modulus, Poisson's ratio, viscosity, nonlinearity, and poroelasticity, to measured mechanical responses. Accurate models are required to predict displacement responses to different mechanical excitations to solve the inverse problem. For simple setups the equations that describe the direct problem have been solved analytically [18]. A similar approach used for irregular domains of elastically heterogeneous tissues is not possible in practice. Consequently, numerical simulations are used to ease this task. Modelling is used in MRE applications in order to create forward models that capture complex mechanisms of wave propagation in soft tissues. Previous studies in this field include various finite difference (FD) [17–19] and Finite Element methods (FE) [15, 20–24]. FE modelling has been used in previous studies for visualization of ultrasonic wave propagation [25–31], elasticity reconstruction [21, 32], and shear wave propagation analysis in gelatin phantoms [33–39].

The paper aims to develop a full three-dimensional (3D) model of shear wave propagation in a gelatin phantom for MRE applications. Some primary investigation has been performed for the bulk wave propagation model based on the Local Interaction Simulation Approach (LISA) [40]. In contrast to the previous work, current investigation focuses on the guided wave propagation with rescaling procedure. The major novelty of the presented work relates to the application of the Local Interaction Simulation Approach (LISA) for guided wave propagation and a rescaling procedure for the LISA is proposed for shear wave propagation modelling. This major novelty is considered to tackle numerical problems.

Then the LISA model is developed to examine density, shear modulus, and shear wavelength in a gelatin phantom. This study proposes the rescaling solution method in order to avoid numerical problems, especially related to wave amplitude. Numerical simulation results are compared with FE simulation results and MRE experimental measurements from a soft tissue mimicking an agarose gelatin phantom.

## 2. Theoretical Background

Elastic wave propagation in an isotropic linear medium is governed by the momentum balance given as

(1) $$\begin{array}{c} \sigma_{ij,i}+b_{i}=\rho \ddot{W_{\iota }}, \end{array}$$

where *σ*_*ij*,*i*_ is the divergence of stress tensor, *b*_*i*_ is an external volume force, and $\ddot{W_{\iota }}$ represents particle acceleration vector. The constitutive equation that relates stresses to strains in a linear elastic solid is given as

(2) $$\begin{array}{c} \sigma_{ij}=\lambda \Delta \delta_{ij}+2\mu \varepsilon_{ij}, \end{array}$$

where *δ*_*ij*_ is the Kronecker delta, Δ represents material dilatation given by Δ = ∇·*W* = *ε*_11_ + *ε*_22_ + *ε*_33_, and *λ*, *μ* represent the Lamé constants for the material. The strain (*ε*_*ij*_) is defined through the strain tensor using the following relationship

(3) $$\begin{array}{c} \varepsilon_{ij}=\frac{1}{2}\left(\;W_{i,j}+W_{j,i}\;\right), \end{array}$$

where *W*_*i*_ represents particle displacement components. Combining (1)–(3) the equation of equilibrium, that is, [41],

(4) $$\begin{array}{c} \mu \nabla^{2}W+\left(\;\lambda +\mu \;\right)\nabla \nabla \cdot W=\rho \ddot{W_{\iota }} \end{array}$$

governs wave propagation in an infinite elastic space and for practical problems must be amended by appropriate boundary and initial conditions describing the problem. Boundary conditions increase the complexity of the problem since they give rise to the so-called guided wave propagation problem, where global wave propagation patterns, that is, modes, travel at different, and possibly frequency-dependent, speeds, as explained in [41]. It is well known that the solution to (4) can be found only for simple canonical problems. Numerical simulations are used for more complex scenarios.

## 3. Numerical Models

This section describes numerical models used for shear wave propagation in soft tissues. Firstly FE model was developed as a reference. Then a LISA model is described. The major focus is on a rescaling procedure that is used to avoid numerical discrepancies.

### 3.1. Finite Element Model

**Figure 1:**
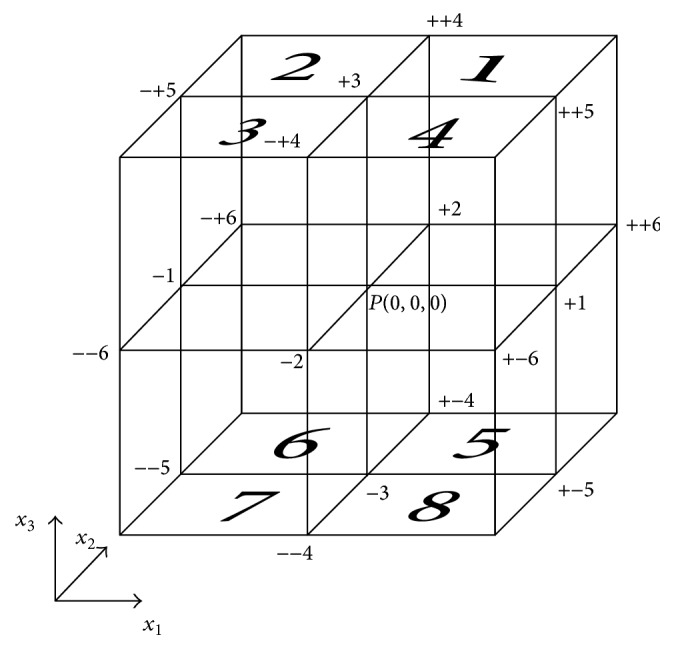
Elementary discretization scheme used for wave propagation modelling in the LISA 3D [42].

The FE model used in the current investigations was developed using the* Marc Mentat 2013* software package. Following the work presented in [36], a 3D cylindrical container, with a diameter of 200 mm and thickness of 20 mm, was modelled using gel phantom material properties. The bottom of the cylinder was fixed in the *y* direction (see Figure 1). Altogether 36 000 elements of 2 × 2 mm radial and axial element size and 200 element along the circle were used. The phantom was modelled as a homogenous isotropic elastic solid with Poisson's ratio *ν* = 0.495. Harmonic sinusoidal motion of 150 Hz was applied to the center of the top cylinder surface as an excitation. Three different elastic moduli (*E*) were investigated, that is, 30, 60, and 120 kPa, to study the relationship between shear wavelengths and shear moduli. Similarly, numerical simulations were performed using three different material density (*ρ*) values, 0.5 × 10^3^, 1 × 10^3^, and 2 × 10^3^ kg/m^3^, for each Young's modulus. Material damping was assumed to be zero.

The shear wavelength (*λ*_*f*_) in the FE model was obtained by estimating the distances between wave peaks directly from response waveforms to make a direct comparisons with the results presented in [36].

### 3.2. Local Interaction Simulation Approach Model

#### 3.2.1. Background of the LISA Model

The LISA, previously used for wave propagation in complex media [42–49], has been applied for MRE shear wave propagation modelling. The algorithm of the LISA model is based on an FD approximation of (4) which discretises any structure under investigation into a grid of cells. Similar discretization is also used in the time domain when modelling is performed. All material properties are assumed to be constant within each cell but may differ between cells. The algorithm can be derived from the elastodynamic wave equation [42]

(5) $$\begin{array}{c} \nabla_{\sigma }\left(\;K\nabla_{\varepsilon }W\;\right)=\rho W_{tt}, \end{array}$$

where *W* = [*u*, *v*, *w*]^*T*^, *W* is the vector of particle displacements, *K* is the stiffness matrix, ∇_*σ*_ and ∇_*ε*_ are the differential operators matrices for stress and strain, respectively, and *ρ* is the density. A comma before the subscript in (5) denotes differentiation. The *K* matrix contains stiffness components *K*_*ij*_^(*p*)^ that depend on Young's moduli and Poisson ratios. The structure is discretised into parallelepiped cells for the 3D LISA wave propagation simulation, as illustrated in Figure 1. The junction of the eight cells characterizes the nodal point *P*. The second time derivatives across the eight cells are needed to converge towards a common value *Ω* at the point *P*. In order to calculate a spatial derivative in the eight surrounding cells to *P*, the central difference scheme is utilized. Then to obtain the solution, stress continuity across adjacent cells is constrained.

The following iteration equations are acquired for each displacement component for a general orthotropic case [42, 49]

(6) χut+1−2ut+ut−1=−2u0∑i,j1,11,2,66,3,551Δxi2·∑p=PKjp+Δx2w0∑p−α1α3PK13+55P+21Δxi∑pPuαiKjP+∑r1,3 ∑pr−2α1α3PwαrrK13−55P+∑p−Pα1α3wα1α35K13+55P+Δx3v0∑p=SPK12+66p+∑p−PSvα1α26K12+66P,χvt+1−2vt+vt−1=−2v0∑i,j1,66,2,22,3,441Δxi2∑p=PKjp+Δx1w0∑p−α2α3PK23+44P+21Δxi∑pPvαiK66,22,44P+∑r2,3 ∑p2r−5α2α3PwαrrK23−44P+∑p−Pα2α3wα2α34K23+44P+Δx3u0∑p=SPK12+66p+∑r1,2 ∑p−1rPSuαrrK12−66P+∑p−PSuα1α26K12+66P,χwt+1−2wt+wt−1=−2w0∑i,j1,55,2,44,3,331Δxi2·∑p=PKjp+Δx1v0∑p−α2α3PK23+44P+21Δxi∑pPvαiK55,44,33P+∑r2,3 ∑p5−2rα2α3PwαrrK23−44P+∑pPα2α3wα2α34K23+44P+Δx2u0∑p=SPK13+55p+∑r1,3 ∑p2−rPα1α3uαrrK13−55P+∑p−Pα1α3uα1α35K13+55P,

where Δ*x*_*i*_ is grid spacing in *i*th direction, *t* is the time step, *α*(*i*) denotes the *i*th column of the *α* matrix of signs, and displacement components are taken at time *t* and point (0,0, 0) if not stated otherwise. The *p* index in the summation formula conveys the sign of the summed component. A detailed derivation of LISA equations can be found in [42]. The local interaction nature of boundary conditions based on matching conditions in the LISA model is the major advantage over the FD-based methods, when used for wave propagation. The so-called Sharp Interface Model (SIM) is used to average physical properties at interface grid points, which represent intersections of four elementary cells. When wave propagation problems in complex media with complex boundaries are studied, the SIM provides more accurate results, as demonstrated in [42–47].

Shear wave propagation in the 3D cylinder, already described in the previous section, was modelled using the LISA approach. Numerical simulations involved the same material properties, boundary conditions, and excitation frequencies as in the FE model described in Section 3.1. These parameters were set following previous investigations reported in [36]. The 3D cylinder was meshed using 1 × 1 × 1 mm elements. Altogether 16 003 000 elements were used in the LISA model.

#### 3.2.2. Rescaling Procedure

**Figure 2:**
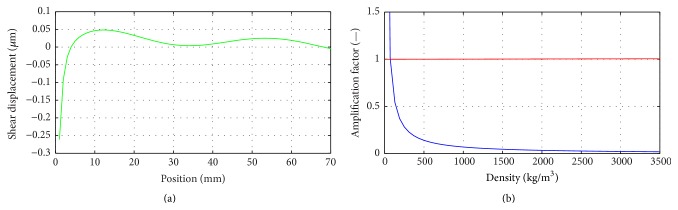
(a) The original shear waveform exhibiting attenuated amplitudes for the density *ρ* = 0.5 × 10^3^ kg/m^3^ at frequency 150 Hz. (b) The amplification factor based on different density.

When a numerical technique is used, such as LISA, for wave propagation simulation, various numerical errors must be accounted for. It is well known that for certain material parameter values elastic waves are quickly damped out making results interpretation cumbersome. This is illustrated in Figure 2(a), where the wave field along the radial direction of the phantom is shown at a single time instant. Clearly, elastic waves are quickly damped out and determination of the wavelength becomes difficult. This problem is a consequence of numerical model properties and can be solved when certain model parameters are modified to avoid numerical discrepancies. Numerical model properties can be investigated in details through the iteration equations analysis. First, the severity of numerical damping can be analysed by considering roots of characteristic polynomial of a numerical scheme at hand, directly related to the Courant-Friedrichs-Lewy stability condition [50]. The latter is frequently invoked in the context of wave propagation modelling as the model parameters are required to meet certain restrictions for the analysis to be stable. This concept can be also used to quantify scheme's accuracy, as will be shown next.

Soft tissues are highly demanding from computational point of view. From physical perspective it is well known that mainly transversally polarized waves propagate in these structures [41]. When numerical modelling is used both types of waves normally coexist. However, analyzing material properties characteristic to soft tissues (these properties are extraordinary when compared to solid media) the difference in longitudinal and shear wave velocities can be immediately found, reaching the ratio of 10. As a consequence, the shear wave component, which is of particular interest for MRE, propagates under conditions far from the stability limit. Namely, the roots associated with the characteristic polynomial drive the waves to decay.

This drawback can be resolved twofold: by reformulating constitutive relationships in order to eliminate the longitudinal wave component, or by manipulating model parameters to push the shear wave closer to the stability limit. In the following work the second approach was employed as this requires no intervention in the solver structure, maintaining the flexibility of the method to model wider class of materials (i.e., solid media and soft tissues).

The longitudinal and shear wave speeds can be expressed

(7) VL=λ+2μρ,VT=μρ.

These definitions show that the density is a parameter that uniformly influences both longitudinal and shear wave velocities. Hence, the approach presented in the paper aims at improving the model properties by rescaling wave speeds. Following the work presented in [36], scaled density is used in numerical simulations. This procedure can be explained using a 1D example of wave propagation. The major focus is on the stability and amplitude accuracy of LISA. The objective of this study is to obtain information about the effect of density on the LISA model. The 1D finite difference equation, involved in numerical simulations, can be expressed as

(8) uin+1=−uin−1+2uin+C2ui+1n−2uin+ui−1n,

(9) C=cΔtΔx,

where *u* is the displacement, *n* is the time step index, *i* relates to the node position, *C* is known as the (dimensionless) Courant (or Courant-Friedrichs-Lewy (CFL)) number, *c* is the wave velocity, and Δ*t*, Δ*x* are related to the time step and the element size, respectively. The stability analysis by means of the Fourier transform is known as the von Neumann analysis [50]. This analysis allows for expression of a governing equation as a recurrence relation that is particularly useful for establishing stability conditions. The key idea is the analysis of the amplification polynomial of the scheme, which is obtained by applying the Fourier transform to the governing FD equation. Once the amplification polynomial is established, certain restrictions are put on its roots. Although the analysis presented is for a 1D case, the entire procedure can be easily extended to provide general stability conditions for higher dimensions.

When (10) is used and the stability condition is obtained for various parameters, stable and unstable conditions can be analysed for various values of density. It is important to note that it is beyond this paper to put all the equation and formula involved in this analysis. Potential readers are referred to [50] for further details. After obtaining the roots of amplification polynomial which are conjugate pairs of the same complex number, the magnitude is the same for both. The magnitude of the roots of amplification factor can be expressed as

(10) $$\begin{array}{c} \left\|\;g\;\right\|=\sqrt{8C^{4}s^{4}-8C^{2}s^{2}+1}, \end{array}$$

where *g* is the amplification factor, *C* is courant number, *s* = sin⁡(*K*Δ*x*/2), and *k* is wave number.

The amplification factor that governs the numerical damping is analysed in Figure 2(b). According to (10), the amplification factor formula, an analysis becomes unstable for *g* > 1, since each consecutive displacement value is increased. Perfect preservation of wave amplitude, that is, without numerical damping, occurs for *g* = 1. For real applications, for example, involving longitudinal and shear waves, *g* is always less than 1 for at least one wave mode; hence waveforms are always numerically distorted. The results shown in Figure 2(b) indicate that when the density decreases, the amplification factor increases (consistently with (7) and (8)) and the instability can be reached in 1D finite difference model.

Numerical simulations of the gel phantom using the LISA approach were conducted and analysis was performed to investigate the effect of density scaling parameter on numerical stability for the 3D case. Various scaling values were selected and respective wave velocities calculated. The initial density was assumed as *ρ* = 0.5 × 10^3^ kg/m^3^. Then, five different scaling parameters were selected as *S*_1_ = 2  (*ρ* = 1 × 10^3^ kg/m^3^), *S*_2_ = 3  (*ρ* = 1.5 × 10^3^ kg/m^3^), *S*_3_ = 4  (*ρ* = 2 × 10^3^ kg/m^3^), *S*_4_ = 5  (*ρ* = 2.5 × 10^3^ kg/m^3^), and *S*_5_ = 6  (*ρ* = 3 × 10^3^ kg/m^3^). The influence of the scaling on model properties is summarised in Table 1. Therein, the limit velocity for the model can be calculated as

(11) $$\begin{array}{c} V_{lim}=\frac{\Delta x}{\Delta t}=\frac{0.001}{0.05e^{6}}=20000\frac{m}{s}. \end{array}$$

Calculation of the amplification factor for a general 3D case is cumbersome; hence it is not provided in the table. However, the general conclusions can be inferred from the CFL numbers as described next.

**Figure 3:**
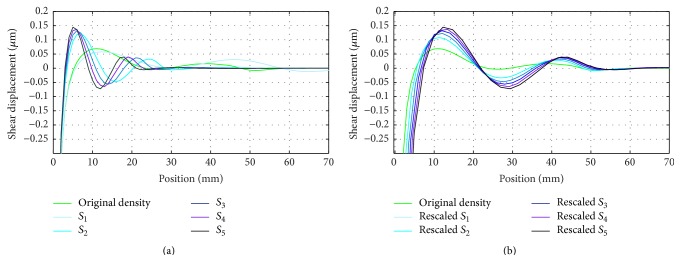
The shear waveform patterns: (a) density-scaled models and (b) after inverse rescaling.

The results in Table 1 show that by increasing the density wave velocities are reduced (see (7) and (8)). As a result the values of the Courant number are also reduced.  Figure 3(a) shows LISA-based simulation results, that is, displacement patterns for different rescaled densities. (Where in the figure horizontal line corresponds to distance of wave propagation form center.)

If the only influence on wave amplitude was due to amplification factor, amplitude drop should have been observed in the simulation. Previous work on the effect of the courant number on pulse distortion in 1D finite difference schemes [51] confirmed these observations. Interestingly, the displacement amplitude increases with the scaling parameter. The latter increase is related to the amount and rate of energy transfer through the excitation. Increased densities result in larger kinetic energies delivered even for unchanged excitations that are prescribed by displacements. This explains the amplitude increase observed in Figure 3(a).

It is clear that once wave propagation is simulated with scaled densities, an inverse spatial scaling procedure should be applied to the results to retrieve proper responses. This is accomplished by an inverse scaling procedure employed for the space sensor waveforms. Again (7) and (8) were employed and space (wavelength) signals were multiplied by the square root of the relevant scaling factors. The results, shown in Figure 3(b), illustrate that the wavelength of the original signal is recovered after the rescaling procedure.

**Figure 4:**
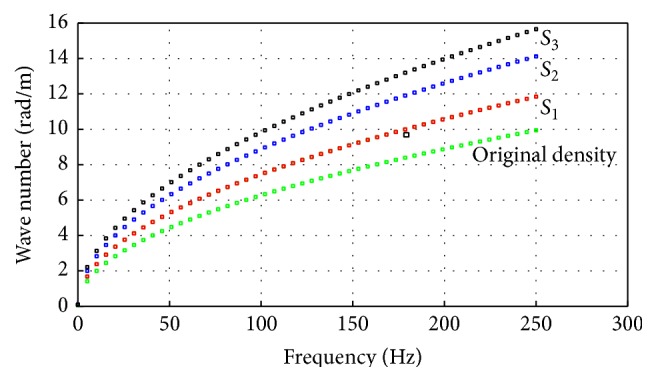
The dispersion plot for the original density and three *S*_1_, *S*_2_, and *S*_3_ scaled densities.

**Figure 5:**
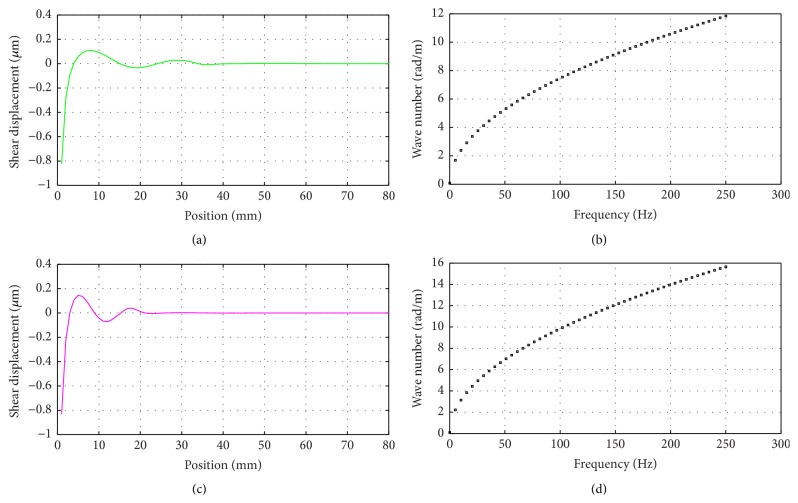
Numerical simulations of shear wave propagation: (a) original waveform (density *ρ* = 1 × 10^3^ kg/m^3^); (b) dispersion curves corresponding to the original waveform; (c) rescaled waveform (density *ρ* = 3 × 10^3^ kg/m^3^); (d) dispersion curves corresponding to the rescaled waveform.

**Figure 6:**
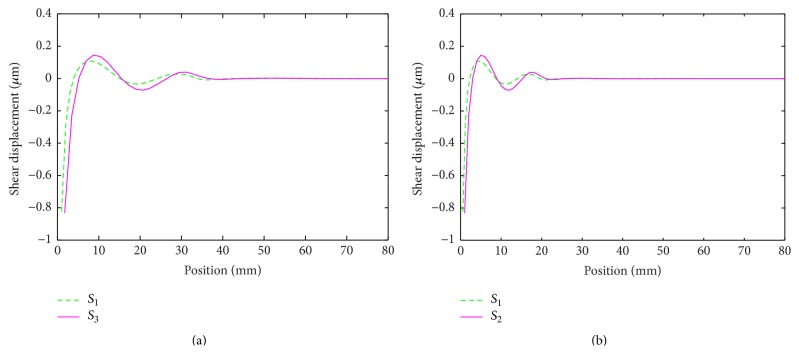
Simulated shear waveforms: (a) after rescaling from *S*_3_ to *S*_1_; (b) after rescaling from *S*_1_ to *S*_3_.

To illustrate the approach, dispersion curves for respective rescaled models were calculated and used to recover the original waveforms. In the following analysis, the *A*_0_ mode is considered, as it is the dominant mode in this frequency range. In Figure 4, dispersion curves for three different scaling parameters are given (*S* in figures correspond by scaling factor). By applying the scaling factor to the original density, it affects the dispersion curve, respectively, which causes a certain change to the wave number of every dispersion curve. The rescaling parameter, square root of *S* – based scaling factor, is also then obtained by analyzing dispersion curves plot between simulated original and scaled density by comparing the ratio of wave number of scaling density by wave number of original density and it proved the efficiency of propose method. To show an example, the waveform (density *ρ* = 1 × 10^3^ kg/m^3^) and one rescaled waveform (*ρ* = 3 × 10^3^ kg/m^3^) together with the corresponding dispersion curves are shown in Figure 5. The wavelength for the original waveform is equal to 21 mm whereas the wavelength for the rescaled waveform is equal to 12 mm. The original waveform (shown in Figure 5(a)) can be recovered from the rescaled waveform (Figure 5(c)) when the latter is multiplied (scaled back) by the square root of the scaling factor (i.e., square root of 3 in this case) and vice versa. The results are shown in Figure 6. The analysis of dispersion curves reinforces the condition that the guided not bulk wave theory should be used in the case investigated, as discussed further in Section 5.

In summary, two interesting observations can be made after the analysis performed in this section. Firstly, the wave amplitude increases when density is rescaled towards larger values. Secondly, the inverse rescaling of waveforms allows one to reproduce accurately the original wavelengths.

## 4. Magnetic Resonance Elastography: Experimental Data

**Figure 7:**
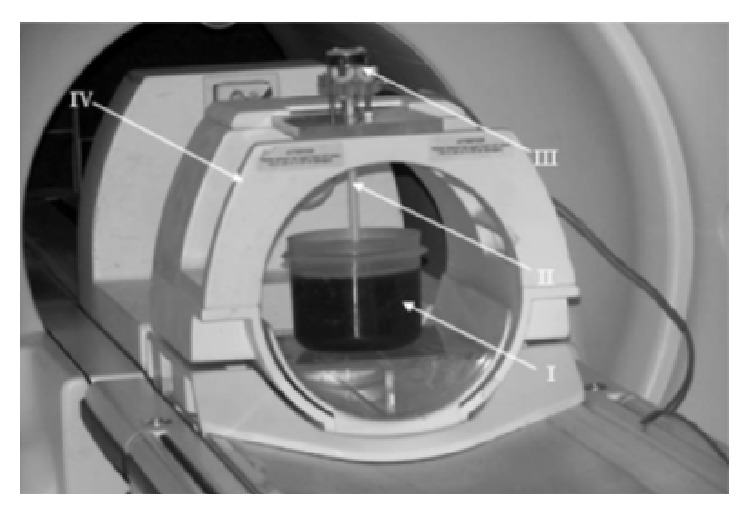
The experimental setup used for MRE tests: I. phantom; II. applicator of the electromechanical driver; III. electromechanical driver; IV. head coil [36].

The MRE data from the experiments reported in [36] were used as a reference in the current investigations. The phantom used in the experiment was a 3D cylinder filled with 2% agarose gel. The geometry of the cylinder was as follows: diameter 150 mm and height 20 mm. The MRE tests were conducted using the 1.5 T* General Electric Signa CT* scanner. The phantom was placed in a head coil and an electromechanical driver was placed on the top surface of the phantom in order to generate shear waves corresponding to the excitation frequency of 150 Hz. The experimental setup used is shown in Figure 7.

The propagation of elastic waves in the phantom was imaged with an MRE pulse sequence sensitive to motion in the horizontal direction. The shear wavelength was estimated manually by calculating the distances between the adjacent wave peaks. Also, the mean of shear wavelength was measured by averaging the wavelength over the four phase offsets. Subsequently, for isotropic elastic infinite solid, an estimate of the local shear modulus *G* can be obtained from the local estimate of wavelength *λ*  as [13]

(12) λ=1fGρ,G=E21+ν.

The shear wavelength *λ* for phantom estimated by MRE was 38.00 ± 2.12 mm at 150 Hz. This corresponds to the mean value of 28.5 kPa for the shear modulus *G*. The shear modulus *G* was also estimated using a dynamic multifrequency shear test with the* DMA 2980* machine for polymer testing to obtain the value of 30 kPa. The density was estimated experimentally as *ρ* = 1.0 × 10^3^ kg/m^3^.

## 5. Numerical Simulation Results

Numerical simulations of shear wave propagation in the phantom described in Section 4 were performed using FE and LISA models. The results are presented in this section.

### 5.1. Shear Wave Propagation in Soft Tissue

**Figure 8:**
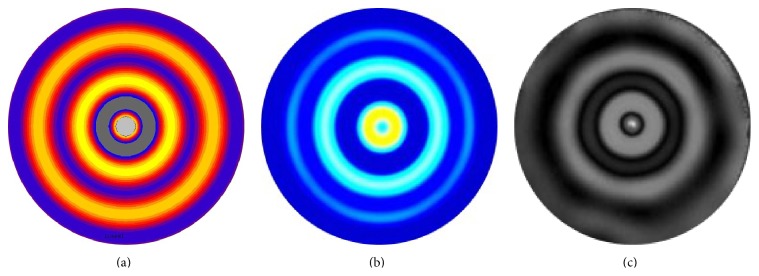
Shear wave propagation patterns for the agarose gel phantom: (a) FE mode [40]; (b) LISA model; (c) MRE experiment [36].

Simulated FE, LISA, and experimental MRE shear wave propagation patterns are presented in Figures 8(a), 8(b), and 8(c), respectively. The simulated results were obtained for Young's modulus *E* = 90 kPa and the density *ρ* = 1.0 × 10^3^ kg/m^3^. The density scaling was applied in LISA models to avoid numerical problems related to excessive wave attenuation. Subsequently, the rescaling procedure was used in postprocessing to recover proper waveforms. The results in Figure 8 show that the simulated and experimental wave patterns reveal the same wavelengths. Small differences between FE and LISA models can be attributed to different formulations of the FE and LISA equations used and differences in meshes.

**Figure 9:**
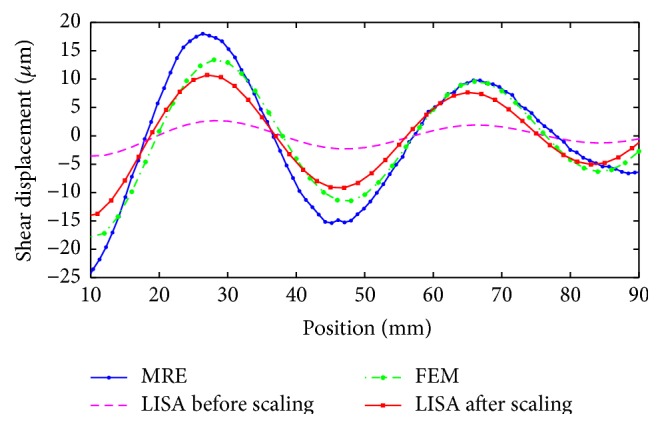
Shear wave propagation, comparison of displacement waveforms for the FE model and LISA model before and after scaling and MRE measurements.

Subsequently, the out-of-plane displacement component responses were acquired from the simulated (FE and LISA models before and after scaling) and experimental (MRE measurements) data. The results, presented in Figure 9, show good agreement between simulated and experimental displacements. It is also important to note that after scaling the amplitude of the LISA model is improved. Next, the shear wavelength was computed from the distance between two successive peaks (or valleys). The wavelengths were estimated as *λ*_*f*_ = 37.5 mm and *λ*_*l*_ = 37 mm for the simulated FE and LISA models, respectively. These results correspond quite well with the MRE-based experimental value of the wavelength *λ*_*m*_ = 38 mm. However, the computational effort of 10 seconds the LISA model compares favorably if compared with the 2640 seconds for the FE model.

**Figure 10:**
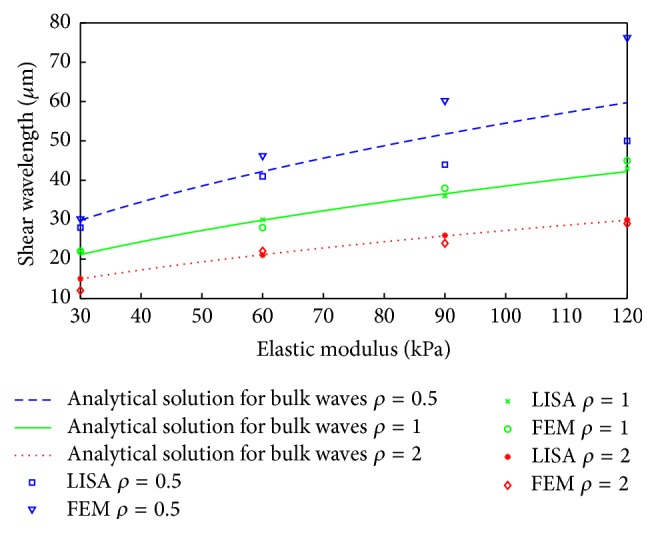
Comparison of shear wavelengths estimated from the FE and LISA models of bulk wave propagation with the relevant analytically estimated wavelengths for different elastic moduli and material densities (0.5, 1 and 2 × 10^3^ kg/m^3^) at frequency of 150 Hz [40].

Following these investigations, simulated shear wavelengths, calculated for different values of elastic moduli and density, were compared with the relevant analytical values calculated from (7) and (8) for the bulk wave propagation problem. Four different elastic moduli, that is, 30, 60, 90, and 120 kPa, and three different densities, that is, 0.5 × 10^3^, 1 × 10^3^, and 2 × 10^3^ kg/m^3^, were investigated.  Figure 10 presents the results for the 150 Hz excitation frequency. Here, the three continuous solid, dashed, and dotted curves give the values of shear wavelengths calculated from (12) for infinite medium propagation model.

Although the results are quite consistent for lower values of elastic moduli, significant discrepancies between numerically (FE and LISA) and analytically (bulk wave propagation solution) estimated results can be observed for higher values of elastic moduli (corresponding to larger wavelengths), particularly for lower densities. These discrepancies are further discussed in the next section.

### 5.2. Guided Wave Propagation in Soft Tissues

Equations (12) provide the relationships between excitation frequency, wavelength, and elastic constants for an infinite elastic space. Thus any estimation of wavelengths, as discussed in the previous sections, and consequently estimation of elastic properties based on these wavelengths is accurate only for the infinite space assumption. This assumption can be approximately fulfilled for the following two conditions: (1) wavelength estimates are made sufficiently far from the object's boundaries; (2) wavelengths are small when compared with distances from the boundaries. Both conditions can be achieved when excitation frequencies are selected to obtain sufficiently short wavelengths. However, near the boundaries wavelength estimations will not be accurate, unless the effect of the interfaces is taken into account.

**Figure 11:**
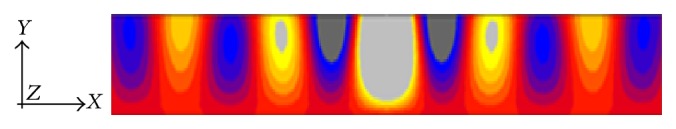
Example of shear wave propagation displacement profile in the phantom.

Figure 11 shows the through-thickness cross-section of the wave propagation displacement field for the analysed phantom model. The results, obtained for the 150 Hz excitation frequency, show that the displacement varies across the thickness of the phantom, from a finite value (top) to zero (bottom). This nonuniform displacement distribution indicates that the wave field is strongly affected by the (top and bottom) boundaries; as a result the (global) wavelength is different from the wavelength for the assumed theoretical infinite space case.

**Figure 12:**
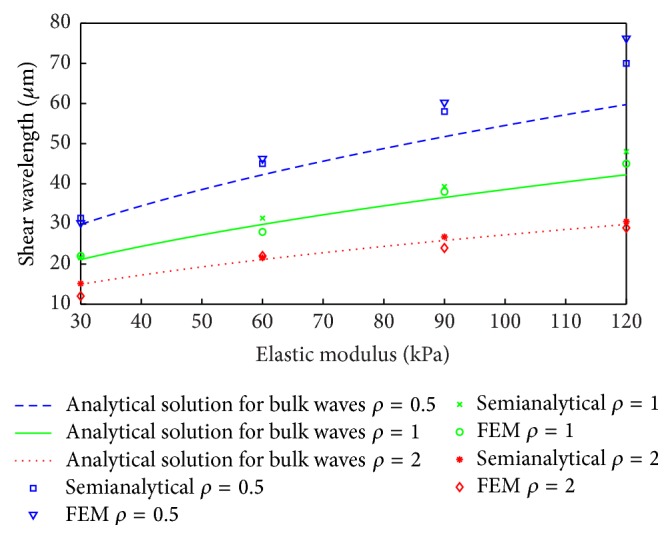
Comparison of shear wavelengths estimated from the semianalytical LISA guided wave model with the relevant analytically estimated wavelengths from bulk wave propagation model for different elastic moduli and material densities (10^3^ kg/m^3^) at frequency of 150 Hz.

These numerically estimated values were compared in Figure 10 with the theoretical values obtained for the assumed infinite space. The results show that the numerical and analytical results start to diverge for wavelengths larger than 20 ÷ 30 mm. This corresponds to the thickness of the phantom. Thus the analysed wave propagation field in the phantom corresponds to the guided wave field rather than to the bulk wave field (assumed in (12)). Guided wave propagation involves partial waves, that is, waves propagating in an infinite space that interact with the (top and bottom) boundaries. These partial waves undergo multiple reflections and mode conversions forming global displacement patterns, that is, wave modes. Therefore, wavelength estimation in the analysed model should involve the relevant dispersion equations for guided waves rather than (7) and (8) for bulk waves. This problem can be solved semianalytically or numerically, as illustrated in [48]. A semianalytical approach, based on the LISA iteration equations, was used in the current paper for wavelength estimation. The vertical (*y*) displacement component at the bottom surface of the phantom was constrained. The results for various Young's moduli and densities are presented in Figure 12. This time, the wavelengths estimated from the guided wave propagation model are compared with the relevant wavelengths estimated from the FE infinite space model (i.e., from (12)). When the results are analysed two distinct wavelength ranges can be distinguished in Figure 12. The semianalytical solution for guided wave propagation compares very well with the bulk wave model for wavelengths shorter than 20 ÷ 30 mm. In contrast, the results for the guided and unbounded media differ significantly for longer wavelengths. The results in Figures 11 and 12 indicate that the guided wave propagation model rather than the bulk wave propagation model (that was originally employed in [36]) should be used for wavelength estimation in the case investigated.

**Figure 13:**
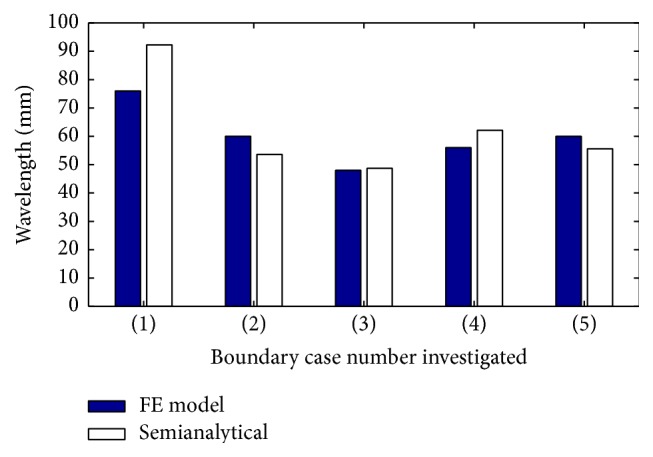
Five different model boundary condition, for both the FE and semianalytical LISA models; (1) 20 mm *y* fixed, (2) 20 mm *x* fixed, (3) 20 mm free, (4) 40 mm *y* fixed, (5) 40 mm free (for Young's modulus *E* = 90 kPa and density *ρ* = 1.0 × 10^3^ kg/m^3^).

Since guided wave propagation is inevitably associated with wave interactions with boundaries, the effect of boundary conditions was investigated. Two different boundary conditions, namely, fixed and free ends, were examined. Altogether five different model scenarios, for both the FE and semianalytical LISA models, were analysed: (1) 20 mm thick phantom with bottom surface fixed in the *y*-direction; (2) 20 mm thick phantom with the *x*-component fixed; (3) 20 mm thick phantom with the free boundaries; (4) 40 mm thick phantom with the *y*-direction fixed; and (5) 40 mm thick phantom with the free boundaries. The relevant calculations were performed for Young's modulus *E* = 90 kPa and density *ρ* = 1.0 × 10^3^ kg/m^3^. All numerically simulated results are presented in Figure 13 and compared with the semianalytical guided wave results. The comparison of the results obtained for the 20 mm thick phantom between the first three model scenarios investigated shows that the estimated wavelength increases when the boundary is fixed. Significantly different responses are obtained particularly for the *y*-constrained direction. This can be attributed to the dominant shear wave propagating in the phantom. In other words analysed displacements are mainly in the *y*-direction and thus the response is more sensitive to this type of boundary condition; thus, a substantial increase in the wavelength can be observed for the model with the *y*-displacement component constrained, as expected.

When the 40 mm thick phantom is analysed, the wavelength is larger for free conditions, if compared with the relevant 20 mm thick phantom in the semianalytical model. The value of wavelength is then further increased by the constraint in the *y*-direction (comparison of scenarios (4) and (5), in the 40 mm case) but an opposite trend was observed for the FE model when boundaries are changed from the free to the constrained in *y*-direction. However, it is important to note the wavelength was calculated in this case from the peak distances and was less accurate than the semianalytical solution to the dispersion relation.

## 6. Conclusions

A 3D rescaled LISA model has been proposed for shear wave propagation analysis. Numerical simulations have been performed to analyze the shear wavelength, that is, the primary parameter characterizing shear modulus, in order to examine several factors that influence shear modulus estimation in homogenous phantoms.

The results show that rescaled LISA can be used very efficiently for shear wave propagation modelling in MRE investigations. Good results agreements have been achieved between the LISA-based, FE model, and experimental MRE measurements. The major advantage of the proposed rescaled LISA method is computational efficiency. Significant reductions of computational effort have been achieved when compared with the classical FE modelling approach. The computational time was reduced more than 260 times for the case investigated.

The results also demonstrate that shear wavelength estimated from the presented LISA and FE models are reasonably close to the theoretical calculations, for homogenous elastic cylindrical phantoms investigated, for shorter wavelengths (i.e, for lower Young's moduli and high densities). In contrast, the solutions based on guided wave propagation are more accurate for longer wavelengths. Also by the rescaling procedure which is presented in this paper, the wave amplitude problems related to numerical errors in soft tissues modelling can be avoided. This analysis can serve as an indicator of interfacial conditions for complex wave propagation in biological tissues.

## Figures and Tables

**Table 1:** The effect of scaled density on wave propagation velocities and numerical stability.

Density	Courant number	Longitudinal wave velocity (*V*_*L*_) and shear wave velocity (*V*_*T*_) [m/s]	Scaling factor in Figures 3(a) and 3(b)	Comments
ρ = 500 kg/m^3^,	$C=\frac{V_{l}}{V_{lim}}=0.3$	*V*_*L*_ = 6.0045*e* ^3^ *V*_*T*_ = 1.4286*e* ^3^	Original density	Green line in Figure 4 relates to initial (original) original density and is used as reference; low amplitudes in numerical simulations

*ρ*_1_ = 1000 kg/m^3^,	$C_{1}=\frac{V_{l1}}{V_{lim}}=0.212$	*V*_*L*1_ = 4.2458*e* ^3^ *V*_*T*1_ = 1.0102*e* ^3^	*S*_1_ = 2	Wavelength was rescaled in Figure 3(b) with the square root of the scaling factor, that is, $\sqrt{2}$

*ρ*_2_ = 1500 kg/m^3^,	$C_{2}=\frac{V_{l2}}{V_{lim}}=0.173$	*V*_*L*2_ = 3.4667*e* ^3^ *V*_*T*2_ = 824.8232	*S*_2_ = 3	Wavelength was rescaled in Figure 3(b) with the square root of the scaling factor, that is, $\sqrt{3}$

*ρ*_3_ = 2000 kg/m^3^,	$C_{3}=\frac{V_{l3}}{V_{lim}}=0.150$	*V*_*L*3_ = 3.0022*e* ^3^ *V*_*T*3_ = 714.3179	*S*_3_ = 4	Wavelength was rescaled in Figure 3(b) with the square root of the scaling factor, that is, $\sqrt{4}$

*ρ*_4_ = 2500 kg/m^3^,	$C_{4}=\frac{V_{l4}}{V_{lim}}=0.13$	*V*_*L*4_ = 2.6853*e* ^3^ *V*_*T*4_ = 638.9053	*S*_4_ = 5	Wavelength was rescaled in Figure 3(b) with the square root of the scaling factor, that is, $\sqrt{5}$

*ρ*_5_ = 3000 kg/m^3^,	$C_{5}=\frac{V_{l5}}{V_{lim}}=0.12$	*V*_*L*5_ = 2.4513*e* ^3^ *V*_*T*5_ = 583.2381	*S*_5_ = 6	Wavelength was rescaled in Figure 3(b) with the square root of the scaling factor, that is, $\sqrt{6}$
